# Navigating childhood fever: A registry-based analysis of parental engagement patterns and information needs in mHealth

**DOI:** 10.1177/20552076261473743

**Published:** 2026-09-07

**Authors:** Moritz Gwiasda, David D. Martin, Ekkehart Jenetzky

**Affiliations:** 1 Institute for Integrative Medicine, Faculty of Health/School of Medicine, 12263 Witten/Herdecke University, Witten, Germany; 2 Department of Pediatrics, Eberhard-Karls University Tübingen, Tübingen, Germany; 3 Department of Child and Adolescent Psychiatry and Psychotherapy, University Medical Center of the Johannes-Gutenberg-University, Mainz, Germany

**Keywords:** mHealth, psychology, fever, patient reportet outcome measures, PROM, digital health, general, FeverApp, fever phobia

## Abstract

**Background:**

Acute childhood fever can lead to parental anxiety which contributes to the overcrowding of pediatric emergency care. Mobile health (mHealth) applications offer support to empower caregivers. Real-world evidence regarding usage disparities and their actual impact on user empowerment remains scarce.

**Objective:**

This observational study investigates the sociodemographic determinants of mHealth usage and evaluates its effect on parental confidence as parent-reported outcome measure (PROM).

**Methods:**

A FeverApp registry data analysis was conducted. An ecological momentary assessment tool regarding fever management of parents. The main sample (N=24,800) was analyzed to identify sociodemographic usage disparities and thematic navigation patterns using correlation clustering. A subsample (n=3,825) completed a 5-point questionnaire regarding perceived safety in managing fever, as the primary PROM. The relationship between interaction frequency and the PROM was evaluated using an ordinal logistic regression model.

**Results:**

Highly educated parents demonstrate the highest engagement (mean = 14.1 interactions) compared to those with lower education (9.8 interactions). The PROM analysis revealed a clear dose-response relationship: higher interaction frequencies strongly predicted higher levels of self-reported safety. Parents with lower educational backgrounds frequently fell below the usage threshold. Furthermore, correlation analysis indicated that parents navigate information in highly structured, action-oriented clusters.

**Conclusions:**

Linking objective data with PROMs suggests that mHealth interventions are positively associated with enhanced parental self-efficacy. The identified equity gap highlights that some designs may leave vulnerable populations behind. To serve as equitable public health tools, future digital health systems must integrate adaptive, low-threshold pathways to ensure the necessary engagement for confident decision-making.

## Background

Even though fever is a fundamentally benign and evolutionary conserved physiological response to infection, serving a crucial role in the body’s immune defense rather than representing a disease entity itself, acute febrile illnesses in children are among the most common reasons for doctor visits worldwide and are one of the main causes of visits to pediatric emergency departments.^1^ While fever is typically a beneficial and self-limiting physiological response to infection, it remains a profound source of parental anxiety a phenomenon widely described as fever phobia.^1^ This phobia extends beyond individual distress and drives significant systemic challenges, including the unnecessary administration of antipyretic medications and avoidable medical consultations.^2^ Ultimately, this uncertainty contributes heavily to unnecessary healthcare utilization of pediatric emergency departments.^2^ Parental anxiety regarding fever management represents not merely an educational gap, but a substantial public health issue that impacts healthcare efficiency.

Consequently, equipping parents with the competence and confidence to manage fever safely at home is not only an individual educational goal but a public health strategy relevant beyond individual reassurance to maintain healthcare system resilience and lower the overcrowding of emergency services.

To recognize the potential of mHealth in acute pediatric settings, it is essential to recognize its established efficacy across the broader medical landscape. Digital health interventions are now utilized in primary and secondary prevention, as well as chronic disease management. For instance, mHealth platforms and smart devices provide critical support for patient self-monitoring in metabolically vulnerable populations, such as in diabetes management,^3^ and serve as vital rehabilitation support for follow-up care after acute events like acute coronary syndrome.^4^ Across these diverse clinical contexts, mHealth has consistently proven its ability to support behavioral change, enhance self-management, and foster patient empowerment. Building upon this robust evidence base, there is growing interest in investigating whether similar mechanisms of digital engagement can also empower parents in the acute management of childhood fever.

The role of mHealth and the need for PROMs in mHealth applications have emerged as promising, scalable tools to bridge the gap between professional care and home management.^5^ By providing 24/7 access to guideline-based information, these applications aim to empower parents in real-time. However, evaluating the actual efficacy of such digital interventions remains challenging. Most studies rely exclusively on objective usage metrics such as log data, download rates, or click frequencies.^6^ While these metrics demonstrate technical feasibility and adoption, they fail to capture whether the interaction actually translates into tangible user empowerment or psychological relief. To truly measure the clinical and emotional value of digital health interventions, objective log data must be systematically linked to patient-reported outcome measures (PROMs). In the FeverApp setting PROMs are providing essential insights into how an app impacts the caregivers’ self-efficacy, decision-making confidence, and perceived safety.^5^

The efficacy of mHealth as a public health solution is further complicated by the “digital divide”. Previous research suggests that mHealth literacy is strongly associated with socioeconomic factors, particularly educational background.^^7^^ If digital interventions are primarily utilized by highly educated parents, they risk exacerbating existing health disparities rather than mitigating them. Despite this risk, there is a lack of large-scale, real-world data analyzing how sociodemographic backgrounds influence actual app usage patterns and, consequently, whether all user groups achieve the necessary engagement thresholds to experience positive PROM outcomes.^8^

To address these gaps, this study analyzes data from the FeverApp, an mHealth app that allows parents to record, track, and manage children’s fever events and symptoms.^9^ By providing scientific information based on current guidelines of the leading pediatric societies in Germany (The Professional Association of Pediatricians and Adolescent Doctors [BVKJ e.V.] and the German Society for Pediatrics and Adolescent Medicine [DGKJ e.V.]), the FeverApp helps parents to better understand and safely manage fever in their child.^8^ The goal of the FeverApp is to create a model registry through families’ self-documentation of fever management through ecological momentary assessment (EMA).^9^ It is crucial to note that the mentioned guidelines explicitly recommend treating the child’s overall discomfort rather than focusing solely on lowering the body temperature. The mHealth intervention investigated in this study aligns with this paradigm by guiding parents to evaluate warning signs and the child’s general well-being, avoiding interventions based strictly on arbitrary temperature thresholds.^10^

By combining large-scale objective usage logs with an integrated PROM assessing parental confidence, this study aims to: (1) Analyze usage disparities by quantifying sociodemographic differences (education, parental role) in mHealth usage intensity and identifying thematic information-seeking patterns via correlation clustering. (2) Evaluate intervention efficacy by determining the relationship between objective interaction frequency and the PROM (self-reported safety in managing fever) to establish whether a “dose-response” effect exists.^11^ (3) Assess digital equity by synthesizing these findings to evaluate whether current mHealth designs provide equitable support across different educational backgrounds.

## Methods

### Study design, data source and population

The present study employs an observational design based on real-world registry data derived from the FeverApp registry. Developed in close collaboration with leading pediatric professional associations, the application was systematically designed to provide evidence-based, guideline-adherent support for parents and caregivers navigating acute childhood fever episodes in a home-care setting. The FeverApp functions as a dual-purpose digital intervention: it offers a real-time documentation interface for tracking body temperature trajectories, accompanying symptoms, and the administration of antipyretic and antibiotic medication, while simultaneously granting users continuous access to a comprehensive medical information library.^10^

The data analyzed in this study were continuously collected under real-world conditions between January 2020 and September 2023, predominantly capturing a user base within the German-speaking region. By utilizing the EMA methodology, the registry captures data with high ecological validity, reflecting actual parental behavior, decision-making processes, and information-seeking patterns during acute moments of medical uncertainty and potential fever phobia.^9^

To address the multidimensional research objectives of this study spanning from the analysis of objective usage mechanics to the evaluation of subjective user empowerment the overarching dataset was methodologically stratified into two distinct analytical cohorts.

The primary cohort, designated as the main usage sample (N=24,800), encompassed all registered users who had generated complete and valid interaction log data within the information library and had provided the necessary sociodemographic baseline information. This extensive, population-scale dataset was utilized to evaluate objective app engagement, quantify sociodemographic usage disparities, such as the digital divide across varying educational backgrounds, and perform correlation clustering to uncover systematic information navigation pathways in an uncontrolled, real-life environment.

The secondary cohort, defined as the PROM Subsample (n=3,825), consisted of a specific subgroup of caregivers who not only engaged with the app’s informational content but also completed an integrated, voluntary evaluation questionnaire. This questionnaire specifically assessed their post-intervention subjective feeling of safety and confidence in managing their child’s fever. Consequently, this targeted subset served as the empirical foundation for evaluating the PROM. This strict stratification allowed for the precise statistical modeling of the dose-response relationship between objective digital engagement (interaction frequency) and the resulting clinical empowerment of the parents, thereby directly linking system usage to a tangible health outcome.

### Data processing and cleaning

The raw app interaction data consisted of server-side log files documenting specific page visits within the information library. To ensure a systematic and valid analysis of these usage data, a multi-step data cleaning process was implemented. In the initial step, technical artifacts such as JSON attachments, specific anchor links, and dynamic URL parameters were removed from the navigational paths to isolate the pure page views. Subsequently, variations of similar URLs and specific subpages were semantically grouped into main thematic categories. For instance, all accesses to subpages detailing temperature measurement techniques were consolidated under a single, unified main category, thereby guaranteeing a consistent count of thematic interactions. A valid interaction was consequently defined as a distinct, cleaned view of a specific information category by a user. The overall usage intensity was calculated as the cumulative sum of all valid interactions per user, while the usage breadth was determined by the number of distinct information categories visited. For the PROM subsample, an additional processing step was required. The cleaned objective usage metrics were precisely linked via pseudonymized identifiers with the subjective responses derived from the in-app questionnaire, which served as the PROM regarding perceived safety. This integration created a comprehensive dataset that allowed the objective clicking behavior to be directly correlated with the perceived empowerment of the parents. To maintain the high data quality of the registry, incomplete datasets, internal test accounts, and log files containing erroneous timestamps or suggested false data entry were systematically excluded from both analytical samples prior to statistical modeling.

### Variables and categorization

To systematically analyze both objective app engagement and subjective outcomes, the study data were categorized into three central variable groups: sociodemographic predictors, objective engagement metrics, and the subjective PROM.

Sociodemographic predictors: Parental educational level served as the primary independent variable to investigate the digital divide. To ensure standardized comparability, self-reported educational qualifications were aggregated into three distinct levels: *High* (university entrance qualification or higher), *Medium* (secondary school certificate), and *Low* (lower secondary school certificate or no formal degree). Additional demographic predictors included the familial role (mother, father) and age, which was divided into four cohorts (<25, 25–34, 35–44, and ≥45 years) to accurately capture age-specific usage disparities.

Objective engagement metrics: Actual app usage was quantified using two continuous variables extracted from the cleaned log data. *Usage Intensity* was defined as the cumulative sum of all valid page views within the information library per user. *Usage Breadth* was operationalized as the total number of distinct thematic categories accessed, reflecting the diversity of the parents’ information-seeking behavior.

Subjective outcome measure (PROM): The primary dependent variable for the efficacy analysis was the PROM regarding perceived safety in fever management. This was measured via an integrated in-app item post-usage and structured as an ordinal variable on a 5-point Likert scale, ranging from 1 (“very low safety”) to 5 (“very high safety”). For the ordinal logistic regression model, this variable was retained in its exact ordered categorical form to optimally align the statistical modeling with the proportional distances of the response options.

### Statistical analysis

All statistical data processing and analyses were performed using the R software environment (version 2026.01.1 Build 403), with the threshold for statistical significance set a priori at α=0.05 for all tests.^12^ To evaluate sociodemographic differences in objective app engagement within the main sample, parametric procedures were utilized, as the large sample size ensures statistical robustness against potential violations of the normal distribution assumption according to the central limit theorem.^13^

For comparisons of continuous usage metrics across multiple independent groups, such as the three educational tiers and four age categories, a one-way analysis of variance (ANOVA) was applied. Independent samples t-tests were utilized for binary group comparisons, such as differences in engagement between mothers and fathers. Chi-Square tests were applied to analyze categorical associations, such as the distribution of education levels within different user clusters.

To identify systematic information-seeking behaviors and understand how caregivers contextually link content, a correlation matrix of the access frequencies across different information library categories was generated.

Using Pearson’s correlation coefficient (r), semantic usage clusters that empirically differentiate distinct navigation pathways were extracted, such as purely diagnostic information seeking versus action-oriented fever management.^14^ Pearson correlation coefficients (r) were calculated between the visit frequencies of different information categories. This allowed for the identification of “usage clusters” groups of topics that are frequently visited together by the same users.

To model the relationship between objective app engagement and subjective empowerment within the PROM subsample (n=3,825), an ordinal logistic regression model was specified.^15^ This non-linear approach is required for the analysis of ordered categorical PROM data, such as the 5-point Likert scale ranging from “very low” (1) to “very high” (5), because it does not falsely assume equal distances between the response categories.^16,17^ The model estimated the log-odds of a caregiver reporting a higher level of perceived safety based on their cumulative number of app interactions, while controlling for educational background to adjust for underlying sociodemographic biases.

To maximize the clinical interpretability of the regression results and to visually evaluate the dose-response relationship, the predicted probabilities for each of the five safety levels were calculated across a continuous range of interaction frequencies using a simulated logit model.^18^ This simulation dynamically illustrates how the probability of reaching maximum safety scores shifts as caregivers intensify their engagement with the digital intervention.

## Results

### Study population and demographics

During the observation period from January 2020 to September 2023, the registry captured data from a highly active user base. After the data cleaning process and the exclusion of incomplete or invalid logs, a total of N = 24,800 users were included in the main usage sample.

To minimize the onboarding burden and respect user privacy during acute caregiving situations, the completion of certain sociodemographic fields such as educational level and specific caregiver role was optional during the registration process. Discrepancies between the total main sample size and the sum of specific sociodemographic subgroups are attributable to these missing responses (e.g., users leaving fields blank, selecting ‘prefer not to say’, or falling into unclassified ‘other’ categories). To maintain transparency, all analyses were conducted using an available-case approach, and the exact valid denominators (n) for each specific variable are explicitly reported in the respective tables.

The demographic distribution showed a strong predominance of female caregivers, with mothers representing 80.8% (n=20,044) of the study population, compared to fathers who made up 16.5% (n=4,101). Regarding age, users were grouped into distinct brackets (<25, 25-34, 35-44, and 45+ years) to evaluate generational differences. The engagement metrics varied in the brackets, with the youngest group (<25 years) showing the lowest engagement and the 35-44 age group showing the highest activity (see Table 1).

**Table 1.** Interactions by user age group.

Age (group)	Users (n)	Age (mean)	Interactions (mean)	Interactions (median)
<25	887	21.1	11.3	5
25-34	11573	30.9	13.0	6
35-44	11029	38.2	14.1	7
>45	1311	50.3	12.3	5

The self-reported educational background of the users exhibited a strong socioeconomic skew. The sample was characterized by a high proportion of academics; the majority of users (70.1%, n=17,398) held a university entrance qualification, classifying them in the high education level. Users with a medium education level (secondary school certificate) accounted for 19.8% (n=4,898) of the cohort. Conversely, parents with a low education level (lower secondary school certificate or no degree) were significantly underrepresented, comprising only 6.3% (n=1,560) of the total user base. The valid denominator for educational level is n=23,856. The remaining n=944 cases represent missing responses from the total main sample.

To evaluate the impact of the app on parental self-efficacy, the confidence subsample of n=3,825 users was extracted from this main cohort. This subset consisted of users who, in addition to using the app, provided specific feedback via the in-app questionnaire regarding their perceived safety and confidence in managing fever. The distribution of safety ratings within this subsample showed that the majority of parents felt empowered by the app, with most ratings clustering in the upper range (Levels 4 and 5).

Regarding content preferences, the analysis of specific category hits revealed a clear hierarchy of information needs. The most frequently accessed categories were “Warning signs”: 22,203 interactions (13.5% of all hits). “What is Fever”: 15,950 interactions.

Administrative or peripheral topics, such as “Sick note”, were visited less frequently (n=7,228), suggesting that the primary driver for app usage is acute medical uncertainty rather than administrative management.

### User engagement by sociodemographic factors

**Figure 1.**
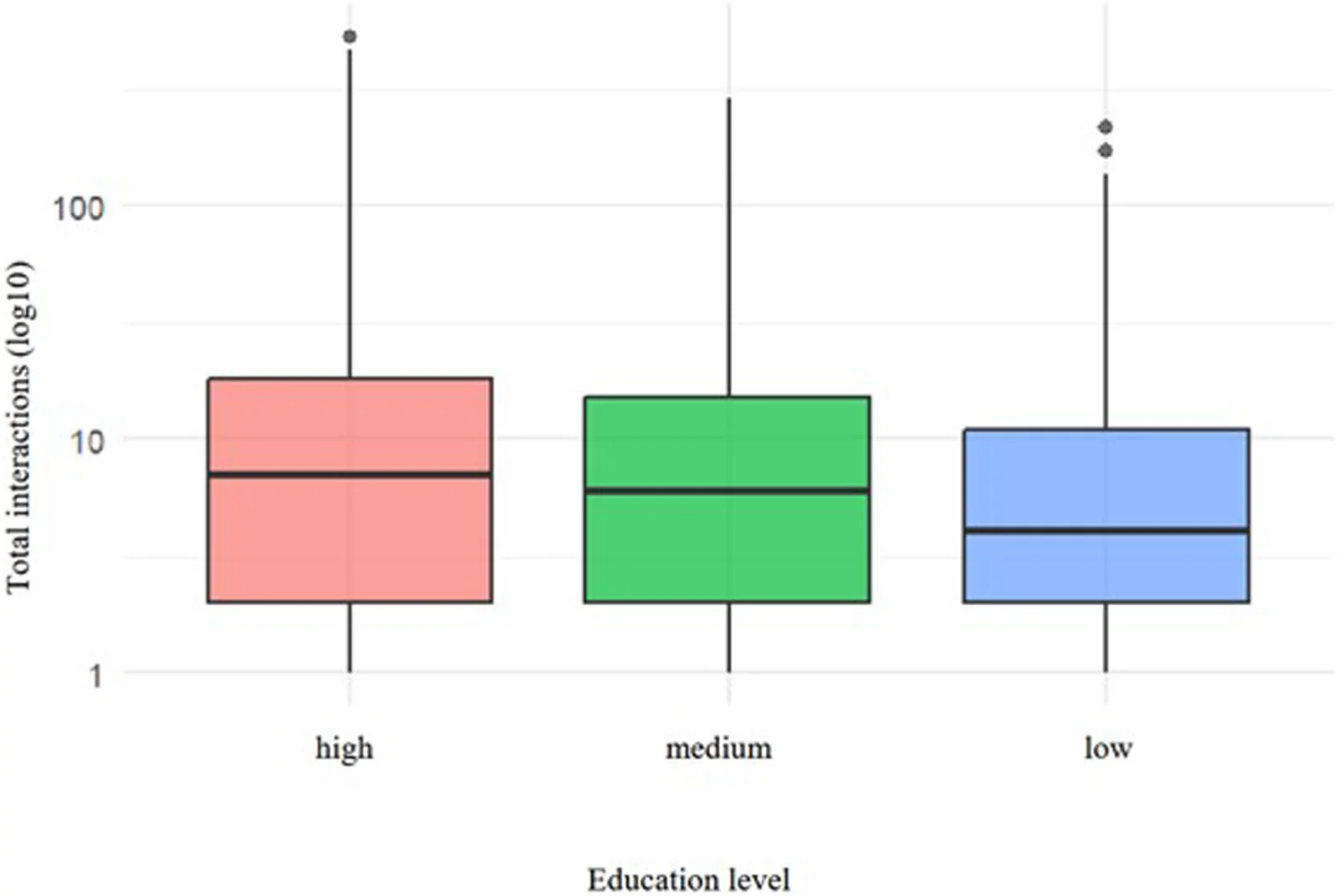
Education level of app users.

Figure 1 shows a clear usage gradient concerning the users’ educational level. Users with a high education level (possessing a university entrance qualification) interacted with the app significantly more often, demonstrating a mean of 14.1 interactions and a median of 7. In contrast, users with a low education level exhibited a mean of only 9.8 interactions and a median of 4. Crucially, this disparity extended to the breadth of information sought. Highly educated parents consulted a wider range of topics, averaging 5.9 distinct categories per user, whereas parents with lower education consulted only 4.3 distinct categories.

Significant differences were also found regarding the parental role and can be seen in Table 2. Mothers emerged as the primary and most active users of the digital intervention, recording a mean of 14.2 interactions and visiting an average of 6.0 distinct categories. Fathers demonstrated significantly lower engagement metrics, with a mean of 9.2 interactions and 4.0 distinct categories. The valid denominator for the specified caregiver roles is n=24,244. The remaining n=556 cases represent “other” roles or missing responses from the total main sample.

**Table 2.** Interactions by parental role.

Category	Users (n)	Interactions (mean)	Interactions (median)	Categories (mean)
Mother	20044	14.2	7	6.0
Father	4101	9.2	4	4.0
Grandfather	44	12.0	7.5	5.6
Grandmother	55	10.1	5	4.5

### Content preferences and usage clusters

During the observation period, the app’s information library was accessed a total of 164,695 times. The analysis of specific category hits revealed a clear hierarchy of parental information needs. The most frequently accessed categories were “Warning signs” with 22,203 interactions, which accounted for 13.5% of all hits, followed by “What is fever” with 15,950 interactions. In contrast, administrative or peripheral topics, such as obtaining a “Sick note for the employer”, were visited significantly less frequently (n=7,228). This suggests that the primary driver for app usage is acute medical uncertainty rather than routine administrative management.

**Figure 2.**
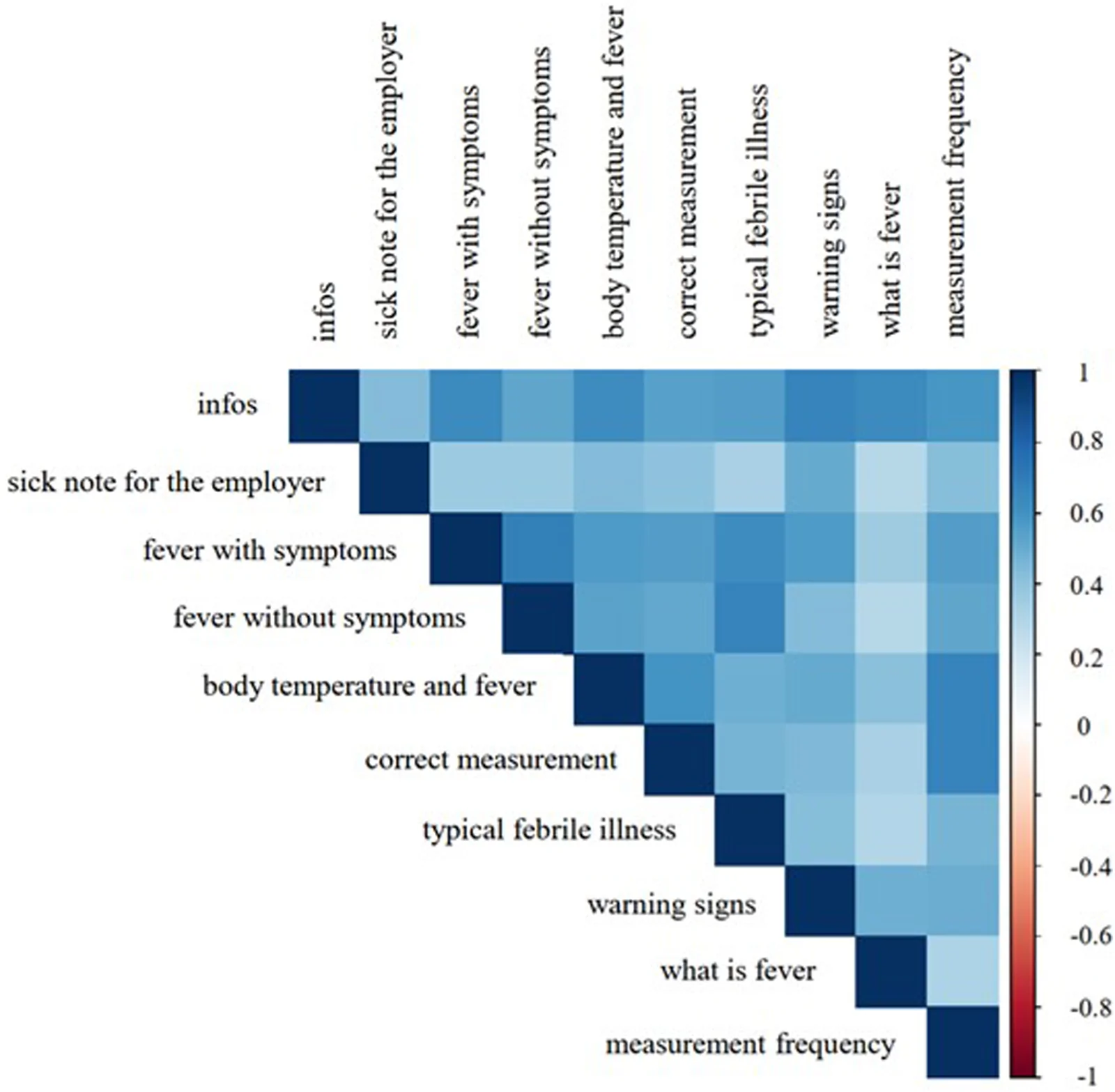
Correlation matrix of the information library categories.

To understand usage patterns and how parents navigate information, a correlation analysis of the visited categories was performed and can be seen in Figure 2. This analysis revealed thematic clusters. The first primary grouping to emerge from the data was the diagnostic cluster. Within this grouping, a strong positive correlation was found between the categories “Fever with symptoms” and “Fever without symptoms” (r=0.68). These categories also correlated strongly with searches for “Typical febrile illnesses” (r=0.66).

Practical instructions formed a second coherent management cluster. The category “Measurement frequency” correlated strongly with both “Body temperature and fever” (r=0.67) and “Correct measurement” (r=0.67). This strong association indicates a high demand among caregivers for direct validation regarding practical, hands-on fever management tasks. Finally, an administrative cluster was identified. Administrative topics, notably the “Sick note for the employer”, showed only moderate to weak correlations with the core medical contents (r<0.50). This distinct pattern suggests that administrative inquiries fulfill a separate, specific user need that occurs independently of the acute medical information-seeking process.

### Association between app usage and parental confidence

**Figure 3.**
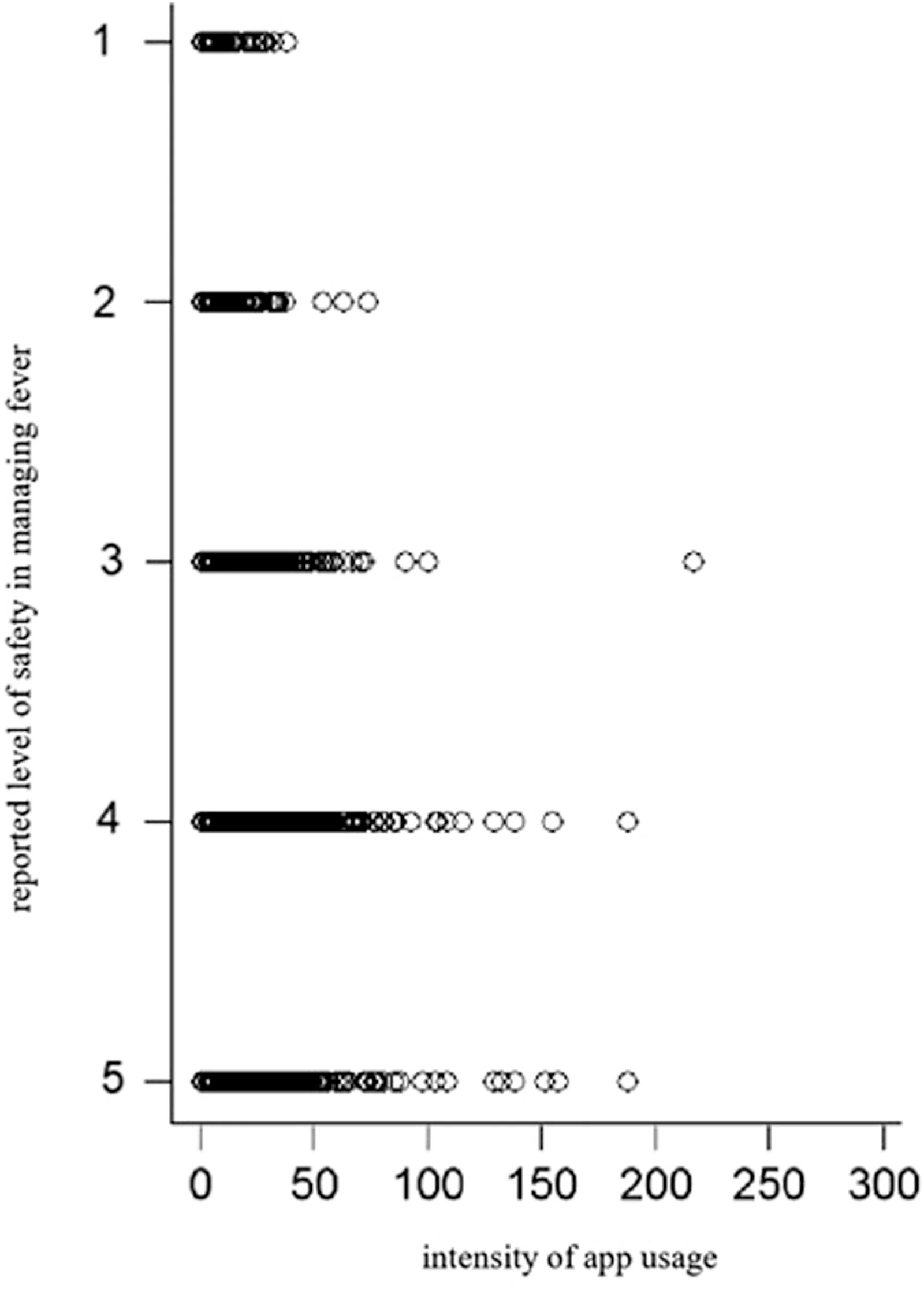
Reported level of safety in managing fever in comparison to app usage.

To assess the clinical impact of the digital intervention on parental self-efficacy, we analyzed the PROM subsample comprising 3,825 users who provided specific feedback regarding their perceived safety in managing fever. The overall distribution of safety ratings indicated that the majority of caregivers felt empowered by the application, with most ratings clustering in the upper efficacy range. A descriptive analysis of engagement by safety level revealed a clear dose-response relationship between the objective intensity of app usage, measured by cumulative interactions, and the subjectively reported level of safety. Figure 3 shows users who reported higher safety levels consistently demonstrated higher average interaction frequencies with the information library. Specifically, the small group reporting very low safety (Level 1, n=75) exhibited the lowest overall engagement with a mean of 8.6 interactions (SD=8.4). Engagement increased marginally for those reporting low safety (Level 2, n=183) with a mean of 9.8 interactions (SD=11.3), and continued to rise for the substantial proportion reporting moderate safety (Level 3, n=877), who averaged 10.8 interactions (SD=14.5). The largest user group, which reported high safety (Level 4, n=1,736), exhibited a marked increase in app usage with a mean of 12.7 interactions (SD=16.2). Finally, parents reporting the absolute highest level of safety (Level 5, n=954) were simultaneously the most active users, recording the highest mean of 14.0 interactions (SD=19.9).

**Figure 4.**
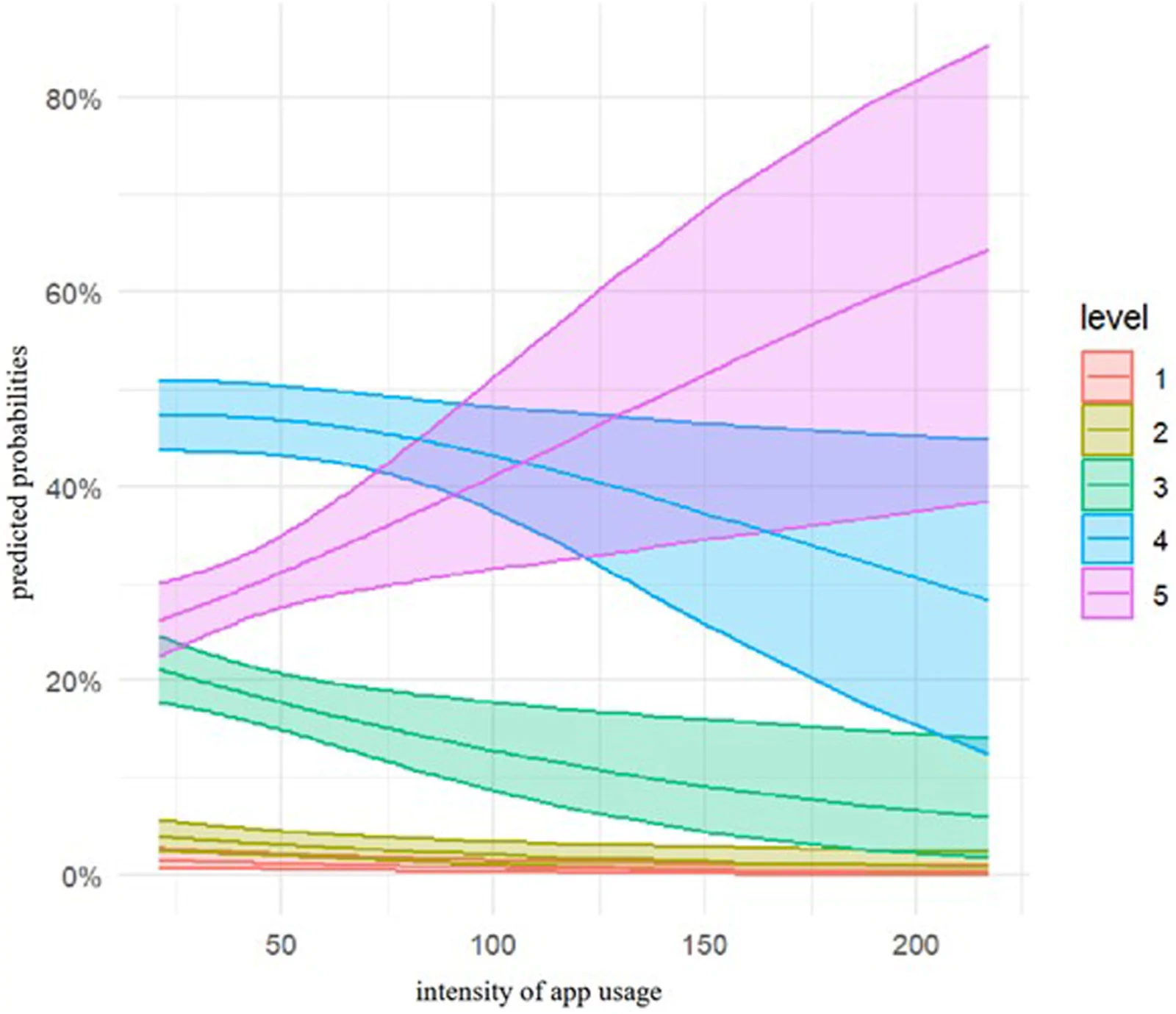
Ordinal logistic regression model of predicted probabilities in safety in fever management.

To statistically confirm this observation, an ordinal logistic regression model was applied, verifying that the cumulative number of interactions acts as a significant positive predictor of higher safety ratings. To visualize this complex effect and enhance interpretability, the predicted probabilities for each safety tier using a simulated logit model was calculated. This simulation reveals a dynamic shift in outcome probabilities as digital engagement intensifies as to be observed in Figure 4. First, the probability of a parent reporting very high safety (Level 5) increases continuously in tandem with the number of app interactions. Second, a saturation shift is observed: the probability of reporting high safety (Level 4) which serves as the dominant baseline category begins to decline at very high interaction levels.

## Discussion

### Study population and demographics

The analysis of the study population’s demographics and corresponding usage metrics reveals a heterogeneous pattern of digital engagement. While mean interaction frequencies provide a general overview of app utilization, the pronounced variance and substantial dispersion in the data highlight a heavily skewed distribution of engagement, the mean is double the median (see Table 1). This dispersion points to the existence of a distinct subgroup of “power users” who interact with the mHealth application with more intensity and frequency, often far exceeding the average interaction thresholds. The presence of these highly active users suggests that while many parents utilize the application for acute, episodic information seeking during a specific febrile event, power users likely integrate the tool as a continuous, comprehensive digital companion for pediatric home care. Understanding this highly engaged demographic is crucial for the interpretation of the data, as they can skew overall usage metrics. Furthermore, they likely represent a particularly health-sensitized, and often highly educated, subset within the broader study population, once again underscoring the need to account for sociodemographic disparities when evaluating the true reach and efficacy of digital health interventions.

### User engagement by sociodemographic factors

While the observed positive association underscores the potential of mHealth interventions to empower caregivers, the observed sociodemographic disparities reveal a digital divide that threatens to undermine these public health benefits. The engagement gradient based on educational background is particularly alarming. Highly educated parents, who inherently possess higher baseline mHealth literacy and greater confidence in navigating text-heavy medical information, utilized the app most intensely and broadly. In contrast, parents with lower educational attainment exhibited significantly reduced interaction frequencies, often failing to reach the engagement thresholds necessary to maximize their self-reported safety. This disparity strongly suggests that current structural designs, which frequently rely on encyclopedic text and self-directed navigation, inadvertently cater to users who are already proficient in processing complex health data. Consequently, digital interventions risk falling victim to the “inverse care law”,^19^ wherein the populations most vulnerable to health disparities and most in need of accessible health literacy support are precisely those least equipped to extract its full benefits.

The data highlights deeply entrenched social patterns in pediatric home care. The predominance and significantly higher engagement intensity of mothers reflect the disproportionate mental load and primary caregiving responsibilities they continue to shoulder during acute childhood illnesses. Fathers, exhibiting notably narrower and less frequent usage patterns, represent a crucial, yet currently untargeted, demographic for digital empowerment. Similarly, the lower engagement among the youngest cohort of parents who objectively possess the least practical parenting experience might suggests that conventional mHealth formats may not align with their preferred digital consumption habits, which increasingly favor multimedia or highly guided algorithmic interactions over self-directed reading.^20^ Addressing this multidimensional digital divide is therefore important for the future development of pediatric mHealth. To ensure that digital applications function as equitable public health tools rather than exclusive resources for socioeconomically privileged groups, developers and researchers must pivot away from one-size-fits-all designs. Integrating adaptive, low-threshold navigational pathways, simplified language options, and proactive guidance will be critical to engaging vulnerable demographics and systematically closing the equity gap in digital health literacy.

### Content preferences and usage clusters

The analysis of content preferences and navigational clusters provides insight into the real-world health information-seeking behavior of parents managing acute childhood illness. The pronounced dominance of interactions with categories such as “Warning signs” and fundamental questions like “What is fever” directly mirrors the psychological core of fever phobia: the uncertainty in distinguishing between a benign, self-limiting physiological response and a potentially life-threatening infection.^2^ By prioritizing warning signs, caregivers demonstrate that their primary objective in utilizing digital health interventions is to establish a safety net and rule out red flags before committing to home-based management.

The identification of distinct navigational clusters indicate that users do not passively or randomly browse mHealth libraries. Instead, parents appear to utilize the application as a dynamic, task-oriented decision-support system, emulating a clinical triage process. The emergence of a dedicated diagnostic cluster where searches for symptoms are connected to queries about typical febrile illnesses illustrates an active attempt by caregivers to contextualize their child’s condition and reduce diagnostic ambiguity. Once this cognitive need for assessment is met, the focus shifts toward the management cluster. The correlation between categories detailing correct measurement techniques and temperature thresholds highlights a substantial demand for practical validation. Parents do not only seek theoretical knowledge, they require actionable, step-by-step guidance to execute caretaking tasks confidently.

The peripheral isolation of the administrative cluster, such as obtaining a sick note for an employer, underscores that organizational requirements are entirely secondary to the immediate emotional and cognitive load of the acute medical situation. These systematic usage pathways suggest that to be truly effective, digital pediatric interventions should not be designed as static medical encyclopedias. Rather, they must be structured to facilitate highly intuitive, action-oriented navigational flows that guide parents seamlessly from initial symptom assessment to practical home-care execution, directly addressing the acute anxieties that could otherwise lead to unnecessary visits to pediatric emergency departments.^9^

### Association between app usage and parental confidence

The association identified between objective app engagement and self-reported parental confidence provides evidence for the clinical utility of mHealth interventions in pediatric home care.

The observed positive association, whereby increased interaction frequencies directly correlate with higher probabilities of caregivers reporting feeling “safe” or “very safe” suggests that digital tools may help mitigate the acute anxieties characteristic of fever phobia.^1^

As digital engagement intensifies as to be observed in Figure 4. First, the probability of a parent reporting very high safety (Level 5) increases continuously with the number of app interactions. Second, a saturation shift is observed: the probability of reporting high safety (Level 4) which serves as the baseline category begins to decline at very high interaction levels. This decline is not indicative of a negative trend; rather, it reflects a saturation effect, wherein heavy users are increasingly likely to graduate from feeling merely safe to feeling very safe. Consequently, extensive engagement with the evidence-based information library appears to be a key driver in maximizing parental confidence and achieving optimal PROM results.

Rather than passively consuming static text, caregivers who engage intensively with the application are utilizing it as a real-time, algorithmic decision-support. By providing evidence-based guidance on critical warning signs, typical illness trajectories, and practical symptom management, the intervention can support diagnostic ambiguity during stressful caregiving moments. This dynamic appears to empower parents to transition from a state of uncertainty to one of actionable competence.^21^ These findings validate the concept of effective engagement within digital health, illustrating that when users achieve sufficient interaction thresholds with structured, high-quality medical resources, they experience a quantifiable improvement in their subjective caregiving capabilities. This psychological empowerment is paramount, as increased parental confidence is a primary mechanism for reducing unnecessary consultations and alleviating the strain on pediatric emergency departments.^2^ Importantly, the increased parental confidence facilitated by mHealth interventions should not be viewed as a replacement for the traditional parent-pediatrician relationship. Rather, tools like the FeverApp are designed to act in addition to professional medical care. By providing immediate, evidence-based guidance during after-hours or acute stress moments, the app can empower parents to manage benign symptoms independently, thereby preserving the parent-pediatrician relationship for cases that truly require clinical evaluation.

### Practical implications for mHealth design

To effectively translate objective engagement into subjective empowerment in mHealth applications across all demographics, future applications should evolve from static, text-heavy information repositories into dynamic, user-centric decision support systems. Because the data indicates that parents naturally navigate information in action-oriented and diagnostic clusters, developers should proactively structure content to mirror these clinical triage processes. Rather than requiring caregivers to independently search and synthesize isolated articles during acute moments of stress, applications should integrate interactive, algorithmic pathways that guide users step-by-step from initial symptom assessment to specific home-care management. This guided approach can reduce the cognitive load and directly addresses the acute uncertainty driving fever phobia.

Furthermore, to bridge the equity gap and effectively reach lower-educated or younger caregivers who currently exhibit suboptimal engagement, mHealth design should prioritize low-threshold accessibility. This involves transitioning away from encyclopedic medical jargon toward simplified plain language and fundamentally rethinking content delivery mechanisms. Integrating rich multimedia formats can bypass the barriers of traditional text-based mHealth literacy. Additionally, implementing proactive design elements, such as targeted push notifications or gamified onboarding processes, could help sustain engagement among high-risk groups, ensuring they achieve the interaction thresholds necessary to generate clinically meaningful improvements in their perceived safety.^22,23^ Ultimately, embedding digital health equity into the core architecture of an intervention means designing not just for the provision of information, but for the active facilitation of comprehension and confident action.

## Limitations

Despite the strengths of utilizing a large, ecologically valid real-world dataset, several limitations must be considered when interpreting the findings of this study. First, the observational nature of the registry data precludes the establishment of strict causal relationships. While the ordinal logistic regression demonstrates a strong, dose-response-like association between interaction frequency and self-reported safety, reverse causality cannot definitively be ruled out. It remains plausible that inherently more anxious parents interact with the application more frequently in search of reassurance, or reversibly not at all to not get confronted with possibly anxiety-inducing information. Rather than the frequency of app interaction independently increasing parental confidence, it is equally possible that a parental baseline level of anxiety dictates their engagement intensity. Consequently, these pre-existing psychological states can influence both the observed usage pathways and the final reported safety levels, making it difficult to determine the true direction of the effect regarding reverse causality.

Additionally, our analysis is subject to residual confounding. Unmeasured variables, such as the child’s underlying chronic health conditions, previous experiences with severe illnesses, or the number of children in the household could simultaneously influence both the frequency of app usage and the caregiver’s baseline confidence levels. The study population can be subject to self-selection bias, a common challenge in mHealth research, which limits the external validity of the results.^24–26^ The registry’s user base is skewed toward highly educated mothers within the German-speaking region. Consequently, fathers, younger parents, and individuals with lower educational attainment or limited baseline mHealth literacy are substantially underrepresented in the main sample. While fever phobia has been documented as a worldwide phenomenon among caregivers across various socio-economic backgrounds, the current study population is drawn predominantly from a German-speaking registry. Future studies should investigate whether the observed navigation patterns and empowerment effects hold true across different cultural contexts and global healthcare systems. This implies that the findings regarding the digital divide, while already statistically significant, may actually underestimate the true extent of the equity gap present in the broader general population. Additionally, evaluating the intervention’s efficacy relied on a single-item Parent-Reported Outcome Measure (PROM) utilizing a 5-point Likert scale to assess perceived safety. Although this streamlined measurement approach is highly suitable for Ecological Momentary Assessment (EMA) because it minimizes user burden during acute, stressful caregiving situations, it inherently lacks the multidimensional psychometric depth of extensive, independently validated mHealth literacy scales.^27,28^ Future research should aim to substantiate these findings through prospective randomized controlled trials and incorporate more granular PROMs to further disentangle the complex mechanisms through which digital interventions mitigate fever phobia.

## Conclusion

By systematically linking objective, real-world engagement metrics with a subjective parent-reported outcome measure (PROM), this study provides empirical evidence suggesting that mHealth interventions may support caregivers and help mitigate the anxieties associated with fever phobia. The identification of a positive association indicates that intensive, structured interaction with an evidence-based digital information library is related to higher levels of perceived parental safety and confidence in home-based fever management. However, the sociodemographic disparities observed in usage intensity reveal an equity gap that threatens the overarching public health utility of such digital tools. The digital divide characterized by the underrepresentation and lower engagement of parents with lower educational backgrounds, younger caregivers, and fathers indicates that current text-heavy, self-directed application designs predominantly benefit those who already possess high mHealth literacy. Consequently, to prevent digital health innovations from inadvertently exacerbating existing health inequalities, a paradigm shift in mHealth development is advised. Future interventions must move beyond a one-size-fits-all design by integrating adaptive, low-threshold navigational pathways and multimedia instructional designs that actively guide users through clinical triage and practical management steps. Ultimately, if digital pediatric systems are to fulfill their promise of reducing unnecessary emergency department utilization and sustainably strengthening health literacy, they must be intentionally engineered to ensure equitable access and engagement for all demographic groups, guaranteeing that no caregiver is left behind in moments of acute medical uncertainty.

## Data Availability

The datasets generated and/or analyzed during the current study are not publicly available due to data privacy regulations but are available from the corresponding author on reasonable request.*
